# Prevalence of childhood obesity and overweight in Bangladesh: findings from a countrywide epidemiological study

**DOI:** 10.1186/1471-2431-14-86

**Published:** 2014-04-01

**Authors:** Tania Bulbul, Mozammel Hoque

**Affiliations:** 1 Center for Communicable Diseases, International Center for Diarrheal Disease Research, Bangladesh, Dhaka, Bangladesh; 2 Departmet of Biochemistry, Bangabandhu Sheikh Mujib Medical University, Dhaka, Bangladesh

## Abstract

**Background:**

Obesity has been declared an epidemic in many high income countries. In low income countries, the coexistence of obesity and underweight makes the situation more grievous. The priority is to explore the overall pictures of body weight status in low income countries and countries that are in transitional phase. Through this country wide cross sectional study we would like to capture the current body weight status among the school aged children, both in urban and rural areas in Bangladesh.

**Methods:**

We conducted a countrywide cross sectional study, from June to September 2009. By random sampling, we selected 10,135 students from 6 to 15 years from both the urban and rural schools. We categorized the students into overweight, obese and underweight by using the values for age and sex at +1SD, +2SD and −2 SD of Z scores of BMI respectively.

**Results:**

We observed among 6 to 15 year olds from both the urban and rural areas 3.5% were obese, 9.5% were overweight and 17.6% were underweight. The proportion of obese and overweight students were greater among the students from urban schools (5.6%, 10.6%) compared to the students from rural schools (1.2%, 8.6%) (RD = 4.3, 95% CI = 3.6, 5.0; RD = 2.0, 95% CI = 0.1, 3.1). The proportion of underweight students were lower in the urban schools (16.1%) compared to the rural schools (19.2%) (RD = −3.1; 95% CI = −4.6, −1.6)

**Conclusions:**

The rate of obesity and overweight is alarming among school aged children in Bangladesh. Overweight and underweight are coexisting which needs special attention to minimize the dual burden.

## Background

The prevalence of obesity is very high in high income countries and many of them have declared obesity an epidemic [1,2]. In the US, obesity is considered a major health problem. Half the population is obese; among adults more than 60% are overweight and more than 30% are obese; among children over 17% are obese [3,4]. In the UK, among adults 23% of men and 25% of women are obese. Among children from 2 to 15 years 5.5% of boys and 7.2% of girls are obese and 22% of boys and 28% of girls are overweight [5].

The low income countries are showing the same trend as the high income countries in increasing rates of obesity. And the duel burden households are very common where underweight and overweight coexist among the children [6]. Rapid urbanization and industrialization are changing the food habits resulting in socio-economic, demographic and cultural changes leading to nutritional transition in low income countries [7,8].

To combat the burden of obesity different stakeholders have taken a variety of strategies to address diet, physical activity and health. Particularly for low-income countries or countries which are in a transitional phase, under-nutrition is still prevalent, and control of obesity will be an additional burden [9].

The proportions of obesity among women in Bangladesh, and the related health and social problems has already been addressed in several studies [10,11]. However, there is a lack of national data for overall body weight status of school aged children. Through this country wide cross sectional study we would like to capture the current body weight status of school aged children both in urban and rural areas in Bangladesh.

## Methods

We conducted a countrywide cross sectional study where we selected school aged children from 6 to 15 year olds from both the rural and urban schools in Bangladesh. We selected 10,135 students using random sampling method. First, we enrolled all six divisions that are the major administrative region of the country. Then we randomly selected two districts those are the second highest tier of regional administrative areas of the country from each division. In the district level we stratified the areas into urban and rural areas. The urban area, a pourasava (municipality) having such amenities as metalled roads, improved communication, electricity, gas, water supply, sewerage, sanitation, and also having higher density of population with majority population in non-agriculture occupations [12]. During selecting a rural area, we selected randomly a sub-district from the selected district (second lowest tier of regional administration). Then from each sub-district we selected a union (the lowest tier of regional administration) randomly.

From each selected union in rural area and municipality in urban area we selected one primary school and one secondary school randomly. Thus in total, we enrolled 48 schools where 24 were primary schools and 24 were secondary schools from 12 urban and 12 rural areas of 12 districts of the country. Based on a previous cross sectional study considered the prevalence of childhood obesity would be close to 7.6% [13]. At the 95% significance level and with 90% power, we would require to enroll 4,007 students. As we enrolled all the students of the selected school, to account for the similarity among the students our estimated sample size was 8,014 would be sufficient considering the design effect of 2 [14]. We collected data from June to September 2009. 36 field workers were recruited and trained to take the body weight and height of the children. A team of three field workers in each school measured the body weight and height of the children at the school premises. The field worker calibrated the weight machine by applying a random set of the standard weights daily to roughly check the accuracy of the weight scales. While measuring the weight the children were asked to put off their shoes. Randomly five boys and five girls from each class from each school were selected to take weight of their school uniform separately and calculated the mean weight of the school uniform by sex. Then we calculated the actual body weight by deducting the mean weight of school uniform from their measured body weight. For taking the height we locally built a stadiometer and checked the horizontal bar was firmly attached with the sliding section before taking the measurement. While taking the height the children were asked to stand straight on bare foot and to look straight in front.

We excluded the child from the study if the child had been suffering from any mental or physical disability or the child was foreigner or from other ethnic minority group. We collected anthropometric measurement (body height and weight) of all the students from the selected schools met the inclusion criteria. We calculated BMI (Body Mass Index) and according to BMI we categorized students into normal weight if BMI was between overweight and underweight with respect to age and sex, overweight if BMI was more than the standardized value for age and sex at +1SD of Z scores of BMI, obese if BMI was more than the standardized value for age and sex at +2SD of Z scores of BMI and underweight if BMI was more than the standardized value for age and sex at -2SD of Z scores of BMI [15].

After getting the approval from Institutional Review Board for the study, the ethical clearance had been obtained from the Ethical Clearance Committee of Bangabandhu Sheikh Mujib Medical University prior data collection. We informed the parents the day before data collection through a letter from the principal that all the students in the school would be interviewed and their height and weight would be measured to check their nutritional status for a study purpose. We obtained informed written consent from the principal or principal nominated teacher where principal was absent. All the students were thoroughly appraised about the nature, purpose and implications of the study and were also assured about their confidentiality and freedom to withdraw themselves from the study any time. All the data were recorded systematically in a preformed data collection sheet. Data were analyzed by using STATA. We described baseline characteristics (age groups, sex). We calculated the proportions of body weight status of the students with 95% confidence intervals. To compare the body weight status among boys and girls, among the students from primary and secondary schools and among the students from urban and rural schools we calculated risk differences with 95% confidence intervals.

## Results

We enrolled total 10,135 students; among them 4,765 students were from rural schools and 5,370 students were from urban schools. In both urban and rural schools the distribution of boys and girls in primary and secondary schools were almost similar (Table 1).

**Table 1:** Baseline characteristics of the enrolled students

	**Rural (N = 4765)**		**Urban (N = 5370)**	
**Total students N = 10,135**	n/N*	**%**	n/N*	**%**
Students from Primary schools				
(age: 6 to 10 years) (N = 4555)	2223/4765	47	2332/5370	43
Boys	1071/2223	48	1229/2332	53
Girls	1152/2223	51	1103/2332	47
Students from secondary schools				
(age: 11 to 15 years) (N = 5560)	2542/4765	53	3038/5370	57
Boys	1229/2542	48	1698/3038	56
Girls	1313/2542	52	1340/3038	44

*n = number of subjects with indicator, N = Total number of subjects.

This study revealed among 6 to 15 year olds from both the rural and urban areas 3.5% (95% CI = 3.0, 4.0) were obese, 9.5% (95% CI = 9.0, 10.0) were overweight and 17.6% (95% CI = 16.0, 18.0) were underweight (Table 2).

**Table 2:** Prevalence of obesity, overweight and underweight among study subjects

**Indicators**	n/N* = 10135	**%**	**95% Confidence interval**
Obese	359	3.5	(3, 4)
Overweight	977	9.6	(9, 10)
Underweight	1780	17.6	(16, 18)

*n = number of subjects with indicator, N = Total number of subjects.

We observed, the proportion of obese students were greater among the students from urban schools (5.6%) compared to the students from rural schools (1.2%) (RD = 4.3; 95% CI = 3.6, 5.0). More students from the urban schools (10.6%) were overweight than the students from rural schools (8.6%) (RD = 2.0; 95% CI = 0.1, 3.1). However, the study revealed the proportion of underweight students was lower in the urban schools (16.1%) compared to the rural schools (19.2%) (RD = −3.1; 95% CI = −4.6, −1.6) (Table 3).

**Table 3:** Body weight status among primary and secondary schools students in urban and rural areas

	**Urban**				**Rural**				
	**Total number of students**	**Primary school students (6 to 10 years)**	**Secondary school students (11 to 15 years)**	**Comparison between primary and secondary students**	**Total number of students**	**Primary school students (6 to 10 years)**	**Secondary school students (11 to 15 years)**	**Comparison between primary and secondary students**	**Comparison between students from urban and rural areas**
Indicators	N = 5370 n (%)*	N = 2332 n (%)*	N = 3038 n (%)*	RD (95% CI)^†^	N = 4765 n (%)*	N = 2223 n (%)*	N = 2542 n (%)*	RD (95% CI)^†^	RD (95% CI)^†^
Obese	300 (5.6)	173 (7.4)	127 (4.2)	−3.2 (−4.4, −2.0)	59 (1.2)	21 (0.9)	38 (1.5)	0.6 (−0.1, 1.2)	4.3 (3.6, 5.0)
Overweight	597 (10.6)	234 (10.0)	333 (11.0)	1.0 (−0.7, 2.6)	410 (8.6)	60 (2.7)	350 (13.8)	11 (9.5, 12.6)	2.0 (0.1, 3.1)
Underweight	865 (16.1)	453 (19.4)	412 (13.6)	−5.9 (−7.8, −3.9)	915 (19.2)	605 (27.2)	310 (12.2)	−15 (−17.2, −12.8)	−3.1 (−4.6, −1.6)

*n = number of subjects with indicator, N = Total number of subjects.

^†^RD = Risk difference indicates the difference of proportions of indicators among the comparison groups. Positive sign (+) denotes, obesity/ overweight/underweight are more common among the children from urban schools than the children from rural schools and more common among the children from secondary schools than the primary schools; negative sign (−) is less common.

While comparing the students by area, among the boys the proportion of obesity was significantly higher in urban schools (5.6%) compared to rural schools (1.5%) (RD = 4; 95% CI = 3, 5). Similarly among the girls the proportions of both obesity and overweight were significantly higher in urban schools (5.5%, 11.1%) compared to rural schools (1.0%, 3.8%) (RD = 7; 95% CI = 6, 9). However, among the girls the proportion of underweight was significantly lower in urban schools (12.3%) compared to rural schools (18.5%) (RD = −6; 95% CI = −8, −4) (Table 4).

**Table 4:** Body weight status among the boys and girls in urban and rural schools

	**Urban schools**			**Rural schools**				
	**Boys**	**Girls**	**Comparison between boys and girls**	**Boys**	**Girls**	**Comparison between boys and girls**	**Comparison among boys between urban and rural schools**	**Comparison among girls between urban and rural schools**
Indicators	N = 2927 n (%)*	N = 2443 n (%)*	RD (95% CI)^†^	N = 2300 n (%)*	N = 2465 n (%)*	RD (95% CI)^†^	RD (95% CI)^†^	RD (95% CI)^†^
Obese	165 (5.6)	135 (5.5)	0.001 (−0.01, 0.01)	35 (1.5)	24 (1.0)	0.01 (−0.001, 0.01)	4 (3, 5)	5 (4, 6)
Overweight	296 (10.1)	271 (11.1)	−0.01 (−0.03, 0.01)	317 (13.8)	93 (3.8)	10 (8.4, 11.6)	−4, (−5, −2)	7 (6, 9)
Underweight	564 (19.3)	301 (12.3)	7 (5, 9)	458 (19.9)	457 (18.5)	1 (−0.01, 0.04)	−0.01 (−3, 2)	−6 (−8, −4)

*n = number of subjects with indicator, N = Total number of subjects.

^†^RD = Risk difference indicates the difference of proportions of indicators among the comparison groups. Positive sign (+) denotes, obesity/ overweight/underweight are more common among the children from urban schools than the children from rural schools and more common among the boys than the girls; negative sign (−) is less common.

While comparing the students by sex, the proportion of overweight among boys (11.7%) was higher compared to girls (7.4%) (RD = 4.3; 95% CI = 3.1, 5.4). The proportion of underweight among boys (19.6%) was higher compared to girls (15.4%) (RD = 4.1; 95% CI = 2.6, 5.6). Among the girls, the proportion of obesity was significantly lower among 11 to 15 years olds (3.8%) compared to 6 to 10 year olds (2.8%) (RD = −1.1; 95% CI = −0.2, −0.1). Among the boys, the proportion of obesity was higher among 11 to 15 year olds (16.1%) compared to 6 to 10 year olds (6.1%) (RD = 9.9; 95% CI = 8.2, 11.7). Among both the boys and girls, the proportion of underweight was significantly lower among 11 to 15 year olds (10.1%) compared to 6 to 10 year olds (21.8%) (RD = −11.7 95% CI = −13.7, −9.7) (Table 5).

**Table 5:** Body weight status among the primary and secondary school students among boys and girls irrespective of area

	**Boys**				**Girls**				
	**Total number of boys**	**Primary students (6 to 10 years)**	**Secondary (11 to 15 years)**	**Comparison among the boys between primary and secondary students**	**Total number of girls**	**Primary students (6 to 10 years)**	**Secondary students (11 to 15 years)**	**Comparison among the girls between primary and secondary students**	**Comparison between boys and girls**
Indicators	N = 5227 n (%)*	N = 2300 n (%)*	N = 2927 n (%)*	RD (95% CI)^†^	N = 4908 n (%)*	N = 2255 n (%)*	N = 2653 n (%)*	RD (95% CI)^†^	RD (95% CI)^†^
Obese	200 (3.8)	108 (4.7)	92 (3.1)	−1.5 (2.6, −0.5)	159 (3.2)	86 (3.8)	73 (2.8)	−1.1 (−0.2, −0.1)	0.6 (−0.1, 1.3)
Overweight	613 (11.7)	141 (6.1)	472 (16.1)	9.9 (8.2, 11.7)	364 (7.4)	153 (6.8)	211 (8)	1.2 (−0.3, 2.6)	4.3 (3.1, 5.4)
Underweight	1022 (19.6)	567 (24.7)	455 (15.5)	−9.1 (−11.2, −7.0)	758 (15.4)	491 (21.8)	267 (10.1)	−11.7 (−13.7, −9.7)	4.1 (2.6, 5.6)

*n = number of subjects with indicator, N = Total number of subjects.

^†^RD = Risk difference indicates the difference of proportions of indicators among the comparison groups. Positive sign (+) denotes, obesity/ overweight/underweight are more common among the boys than the girls and more common among the children from secondary schools than the primary schools; negative sign (−) is less common.

## Discussion

The prevalence of obesity and overweight among Bangladeshi school children of 6 to 15 year olds found 3.5% and 9.7% respectively. The available data from the other South East Asian showed the similar rate of obesity among school aged children [16]. Among the Saudi children from 6 to 18 year olds the prevalence of obesity and overweight was around 6% and 12% respectively [17]. Though the rate of obesity is higher in high income countries, the rate of obesity is also increasing in low income countries. Most studies from the high income countries reported an increasing prevalence of obesity in childhood and adolescence period [18]. However, in Thailand, obesity among 6 to 12 year olds increased from 12.2% to 15.6% in two years, between 1991 and 1993 [19]. A recent cross sectional study conducted in Dhaka city among 5000 study subjects identified 380 children (7.6%) were obese [13]. The proportion of obesity from this previous study was much higher probably due to smaller sample size and also they enrolled a subgroup of population from a metropolitan city in contrast to our countrywide sampling.

There is good evidence that the rate of childhood obesity is increasing in low and middle income countries. Malnutrition and obesity coexist in many low income countries. According to a review article, obesity does not appear to be public health problem among school children in Asia and sub-Saharan Africa. However, many countries in Latin America, Caribbean, Middle East, North Africa, central/Eastern Europe and the common wealth of independent states, the proportions of obesity among children are as high as they are in United States [7,20]. Considering the large differences in socio-cultural contexts of these countries and rapidity of the epidemiologic transition, the extent of childhood obesity and overweight largely differs across countries [2].

Our study revealed high level of underweight (17.6%) among 6 to 15 year olds. An analysis of 160 nationally representative surveys from 94 low income countries showed an increasing rate of overweight and obesity among the obese children from childhood to adulthood, although rates of early childhood malnutrition remained relatively high. Certain countries demonstrated high prevalence of overweight in conjunction with high frequencies of malnutrition. In northern Africa, the proportion of overweight children exceeded 8% and that of children with wasting exceeded 7%. Similarly in south east-Asia, 2.4% of preschool children are overweight and 10.4% suffers from wasting [21]. However, another review article with based on nationally representative data from Brazil, China, United States of America and Russia showed the inverse relationship of co-existence of overweight and underweight [22].

This study revealed, more boys were both overweight and underweight compared to girls among 6 to 15 year olds. A cross sectional survey in Lebanon showed prevalence of childhood obesity much higher in boys (7.5%) than the girls (3.2%) among 3 to 19 year olds [23]. Another study on the Finish children observed greater rate of overweight and obesity among the boys than the girls [24]. In USA, boys were more at risk for overweight than the girls among 6 to 19 year olds [3]. However, the sex distribution varies among the ethnic groups. Another study in USA showed more girls were overweight and obese among the non-Hispanic black children than the boys among 12 to 19 year olds [25].

Compared to rural children among 6 to 15 year olds, the study revealed more children in the urban schools were overweight and obese. From a cross-sectional study was performed on 541 children (273 boys and 268 girls) aged 6–7 years, in urban and rural areas of South-East Serbia found more obese children in urban areas than the rural areas. This difference ascribed to more sweets and fast food consumption and spending time in watching TV among the urban children compared to rural children [26]. Another study in China showed that the rate of childhood obesity is more in urban children (23-30%) than in rural children (16-20%) and the difference was not only because of that the children in rural China are physically more active but also the reason is the urban children consume more fat than rural children. Urban children in China are engaged in more academic work because they are under pressure to achieve scholastic excellence, whereas rural Chinese children are engaged more in physical work to meet economic necessity [27].

Lower to middle income nations face double burden of having both malnourished and over nourished populations, where overweight and obese children being concentrated in urban areas mostly. In developing countries, the rapid progress of urbanization is associated with a cluster of non-communicable diseases and unhealthy lifestyles, which results in very high rates of obesity and its consequent morbidity and mortality. Moreover, in such communities, childhood obesity is still considered a sign of healthiness and high social class [28].

## Conclusions

The prevalence of obesity and overweight is alarming among the school aged children in Bangladesh. While underweight is still predominating among the rural children, obesity and overweight prevailing among the urban children. To combat the future dual burden of communicable diseases and the non-communicable diseases as a consequence of underweight and overweight, we need to understand the social perspective of overweight and obesity in the low income countries in order to take appropriate measure by the government and other stakeholders.

## Competing interests

The authors declare that they have no competing interests.

## Authors’ contributions

TB conceptualized, designed, analyzed data and wrote the manuscript. MH contributed in study design and writing. And also have given final approval of the version to be published. Both authors read and approved the final manuscript.

## Authors’ information

Tania Bulbul, MD, MPH, Assistant scientist, CCD, icddr,b, Dhaka, Bangladesh

Dr. Md. Mozammel Hoque, Professor, Department of Biochemistry, Bangabandhu Sheikh Mujib Medical University, Dhaka, Bangladesh.

## Pre-publication history

The pre-publication history for this paper can be accessed here:

http://www.biomedcentral.com/1471-2431/14/86/prepub
